# Structural Rearrangements of Carbonic Anhydrase Entrapped in Sol-Gel Magnetite Determined by ATR–FTIR Spectroscopy

**DOI:** 10.3390/ijms23115975

**Published:** 2022-05-26

**Authors:** Vladimir Ivanovski, Olga E. Shapovalova, Andrey S. Drozdov

**Affiliations:** 1 Faculty of Natural Sciences and Mathematics, Institute of Chemistry, Ss. Cyril and Methodius University in Skopje, Arhimedova 5, 1000 Skopje, North Macedonia; 2 SCAMT Institute, ITMO University, Lomonosova St. 9, 191002 Saint Petersburg, Russia; shapovalova@scamt-itmo.ru; 3 Moscow Institute of Physics and Technology, Institutsky Ave. 9, 141701 Dolgoprudny, Moscow Region, Russia

**Keywords:** magnetite, sol-gel, carbonic anhydrase, protein secondary structure, entrapment, infrared spectroscopy

## Abstract

Enzymatically active nanocomposites are a perspective class of bioactive materials that finds their application in numerous fields of science and technology ranging from biosensors and therapeutic agents to industrial catalysts. Key properties of such systems are their stability and activity under various conditions, the problems that are addressed in any research devoted to this class of materials. Understanding the principles that govern these properties is critical to the development of the field, especially when it comes to a new class of bioactive systems. Recently, a new class of enzymatically doped magnetite-based sol-gel systems emerged and paved the way for a variety of potent bioactive magnetic materials with improved thermal stability. Such systems already showed themself as perspective industrial and therapeutic agents, but are still under intense investigation and many aspects are still unclear. Here we made a first attempt to describe the interaction of biomolecules with magnetite-based sol-gel materials and to investigate facets of protein structure rearrangements occurring within the pores of magnetite sol-gel matrix using ATR Fourier-transform infrared spectroscopy.

## 1. Introduction

Enzymatic nanocomposites and hybrid materials are emerging classes of bioactive materials which attract a vast amount of attention due to the potential contained in such systems for a variety of applications. Enzymatic composites are intensively studied for the purposes of industrial applications, analytics, smart materials, and next-level nanotherapeutics [1,2,3,4,5]. Such composites may be synthesized in a variety of ways depending on the proposed application scenario, but among them, special attention is paid to sol-gel composites. Sol-gel materials have shown themselves as excellent carriers of small biomolecules, proteins, and even whole cells by the so-called entrapment strategy [6,7,8]. This approach is based on the process of in-situ gelation of the corresponding sols in presence of corresponding dopants, which become physically trapped within forming gel structures. The high porosity of the resulting composites ensures steady mass transfer inside such systems allowing trapped species to maintain their catalytic activities and at the same time preventing them from leaching into surrounding media due to sterical reasons [9,10]. Moreover, it was reported that incorporation of biopolymers into the porous structure of the final material may enhance their stability towards numerous destructive factors, such as high temperature or oxidizing agents [11,12]. As the result, many bioactive systems were described, such as highly stable biosensors, industrially important biocatalysts, or even cell-based implants [13,14,15].

The imposed stability of the immobilized agents is implemented by several factors. First of all, encapsulated biomolecules are protected from biodegradation by enzymes or microorganisms. Second, it should be taken into consideration that the gel microenvironment can be highly hydrated due to water molecules tightly associated with the material of the porous matrix [16]. And lastly, the structure of biomolecules can be stabilized by the form of the pores themselves, densely surrounding the dopant. These effects can be even further improved by the addition of osmolytes or hydrophilic agents, resulting in highly stable systems which can be stored on a shelf at room temperature for long periods without degradation, giving rise to a variety of potential application scenarios of such systems [17]. The majority of sol-gel systems are based on silica as it was the first and best-studied sol-gel material suitable for entrapment, while in recent years several more systems were invented including alumina, titania, zinc oxide and others [18,19,20,21]. Among them, a special place should be given to magnetic sol-gel systems based on iron oxides due to the unique magnetic properties of such materials [22,23,24]. Magnetic enzymatic sol-gel systems are interesting not only due to their ability to be localized or extracted with constant magnetic fields but also due to their sensitivity to alternating radio-frequency fields which can be used for heating those materials and control their catalytic activity, allowing to couple physical stimuli and biochemical reactions [25]. It should be noted that magnetite-based matrices are the most recent types of sol-gel materials used for enzyme entrapment and are under intense investigation. While the functionality of such systems was thoroughly studied it has never been investigated from a fundamental point of view and the observed unique properties were observed and described but not understood. There are several examples in the literature devoted to the investigation of proteins within sol-gel matrices using FTIR and/or circular dichroism spectroscopies, describing the fate of biomolecules in the constrained environment of sol-gel pores and the occurring transformations in their structure [18,26,27,28], but it was never done for magnetite-based systems.

Here, to the best of our knowledge, we make the first attempt to look into the unique features of magnetite-based enzymatic sol-gel composites and explain the previously observed increased thermal stability of proteins entrapped within magnetite sol-gel matrix. We took the carbonic anhydrase enzyme as a model and investigated alternations in its structure by careful analysis of the hybrid material with Fourier-transform infrared spectroscopy (FTIR) at various temperatures. By circumscribing the alternation in the entrapped enzyme’s secondary structure, we are giving our vision of the processes taking place inside the pores of the magnetic bioceramic and shedding light on the fate of the protein inside the opaque matrix.

## 2. Results

**Figure 1:**
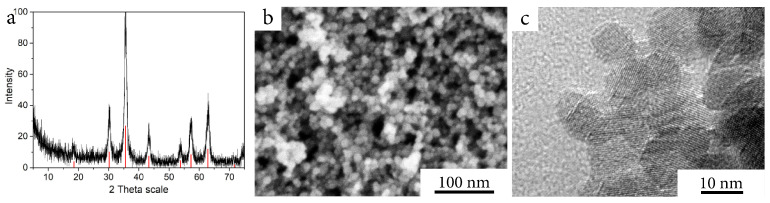
Magnetite nanoparticles: (**a**) XRD pattern of the synthesized material. (**b**) SEM image of the material. (**c**) TEM image of the material.

Ferria hydrosol used in this study was synthesized via an ultrasonically-assisted co-precipitation synthetic strategy that gives a stable hydrosol of magnetite NPs in water at a neutral pH level. The produced magnetite nanoparticles had a mean diameter of 10 nm according to XRD, SEM, and TEM analysis with a truncated tetrahedron morphology, typical for co-precipitation synthetic procedures (Figure 1a–c), while the application of non-stochiometric ratio of iron (II) and (III) ions led to the enrichment of the NPs surface with Fe(II)-OH groups that shifted their isoelectric point to higher pH level of 8.2–8.4. As the result, the system had a highly positive zeta potential of +31 mV, ensuring their colloidal stability at neutral or slightly basic pH levels and in the presence of low salt concentrations. The absence of any modifiers on the surface, such as surfactants or citrate ions, allowed such NPs to undergo sol-gel transition after partial solvent removal, resulting in stable mesoporous sol-gel matrices (Ferria) via the formation of Fe-O-Fe inter-particle bonds [29]. This fact allowed to use such matrices for the entrapment of proteins or enzymes under mild conditions.

**Figure 2:**
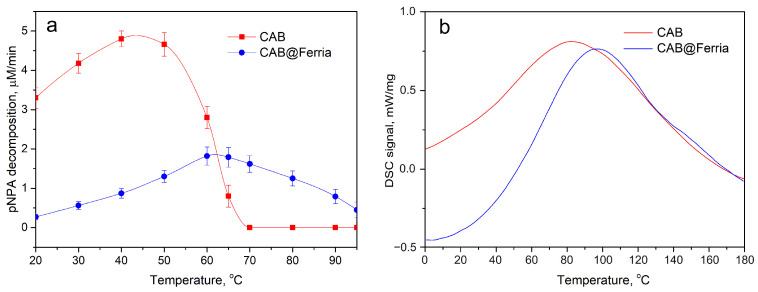
Thermal behaviour of free CAB and CAB@Ferria: (**a**) Catalytic activity of free CAB and CAB@Ferria as a function of temperature. The points represents average of three experiments. (**b**) DCS curves of free CAB and CAB@Ferria.

The porous structure of sol-gel Ferria ensured mass transfer within the material and allowed entrapped CAB to retain its catalytic activity at the level of 8% of its initial values at 20 °C. At the same time, the immobilized enzyme showed significantly higher thermal stability and preserved its enzymatic activity even at 95 °C, while the free enzyme totally denatured at 70 °C (See Table 1 and Figure 2a). The composite material may be active even at higher temperatures, but the boiling of water made the experiments hard to analyze. It can be seen, that T${}_{opt}$ for hybrid material is shifted approximately 20 °C higher compared to free enzyme due to stabilizing effect of 3D matrix and elevated mass transfer processes in the porous material.

Enhanced thermal stability of immobilized CAB can be seen from DCS curves of the free enzyme and CAB@Ferria (Figure 2b). The peak corresponding to maximal heat consumption corresponding to the biomolecule’s structure rearrangement and its denaturation is shifted for 30 °C to a higher temperature, proving enhanced stability of the immobilized enzyme.

More detailed investigations of the occurring rearrangements were done using infrared spectroscopy. It is known from the literature that secondary structure elements of proteins has different sensitivity to to elevated temperatures and typically helical structures are more affected than β-sheet [30]. For instance, β-lactoglobulin at temperatures of 70 °C lose almost all α-helix and one-fifth of the β-sheet compared to the native structure, forming so-called molten globular state [31]. This molten globular state also was observed in the work on Bovine Carbonic Anhydrase (CAB) [32], where the changes in the structure of the protein with the temperature at pH 2.6 were discussed. It appears that raising the temperature to 67 °C, one-third of the β-sheet was lost in a non-cooperative way, while the rest, until 87 °C in a cooperative manner. The percentage of β-sheet in CAB as reported in [32] is ca. 40%. The more precise measurements using FTIR, X-ray, and Circular Dichroism [33], report this percentage to be 49%, while α-helix 13%. In all the above references, the Amide I band was investigated in order to establish the percentage of secondary structures, but also to investigate its intensity and frequency change with temperature [30,31,32]. As proposed in Ref. [33] and further supported in [34], the Amide I band of CAB can be deconvoluted in 8 bands: 1625, 1636 and 1678 cm${}^{-1}$ β-structures, 1653 cm${}^{-1}$ helices, 1645 cm${}^{-1}$ unordered structure and 1660, 1668, 1690 cm${}^{-1}$ turns and bends. Apart from an investigation of proteins in D${}_{2}$O solutions, an analysis of thin dry films of Bovine Serum Albumin as a function of temperature was performed in Ref. [35]. As our samples were dry CAB and also dried CAB@Ferria, we adapted this approach taking advantage of the fact that IR-ATR can be used for spectra recording. We were not using the approach presented in Ref. [36] of calculating the absorbance spectrum from recorded ATR using Kramers-Kronig transformation, but instead directly converted ATR reflectance into absorbance spectrum, which for the conclusions of this work appeared satisfying.

**Figure 3:**
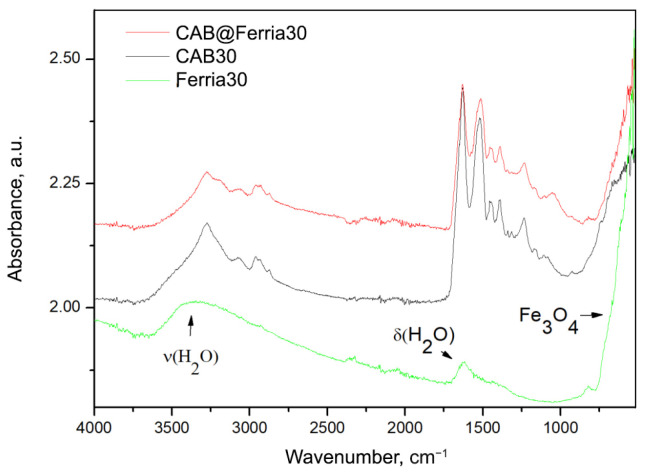
ATR converted to absorbance spectra of CAB@Ferria, CAB and Ferria at 30 °C. The bands on Ferria spectra are assigned are marked with arrows.

The transformed ATR spectra of CAB, CAB@Ferria, and Ferria are presented in Figure 3. It is possible to see that in the spectrum of Ferria, apart from the bands around 570 cm${}^{-1}$, characteristic for Fe${}_{3}$O${}_{4}$, bands due to absorbed water are also present [37].

**Figure 4:**
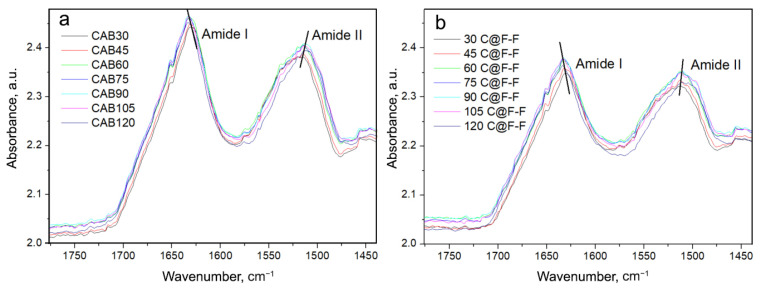
(**a**) ATR converted spectra of native CAB recorded at different temperatures. (**b**) CAB spectra were obtained after subtraction of CAB@Ferria and Ferria spectra (named C@F-F), at different temperatures with a minimized difference. Amide I and Amide II frequency regions are presented. The lines indicate the tendency to shift the maxima of Amide I and Amide II bands with the temperature change.

In order to be able to compare the native CAB spectra with the spectra of CAB which was entrapped in the Ferria cage, we subtracted the spectrum of Ferria from the spectrum of CAB@Ferria at each temperature, respectively. Since the penetration depth of the radiation for different samples is different, which affects the absorbance band, a program was written in Mathematica program package that minimized the deviation between subtracted CAB@Ferria and Ferria spectra and the corresponding CAB spectrum. The spectra of CAB and the CAB@Ferria minus Ferria spectra (in the further text C@F-F), at different temperatures, are presented in Figure 4.

One obvious feature in Figure 4 is the shift of the Amide I band to higher frequencies and Amide II band to lower frequencies with the increase in temperature. This might be due to the weakening or breaking of hydrogen bonds in the protein [35]. Since Amide I band appears as a result of primarily ν(C=O) vibration, while Amide II is mostly due to ν(N-H), the above effect is obvious and appears in both CAB and C@F-F spectra. The range of the shift, in both cases, is about 2 cm${}^{-1}$, for both Amide I and II bands. However, one difference between CAB and C@F-F spectra is that the maximum of the Amide II band at 30 °C for the former is located at 1513 cm${}^{-1}$, while for the latter at 1510 cm${}^{-1}$. Another difference is that at 120 °C, the Amide II shoulder is present at 1497 cm${}^{-1}$ in the C@F-F spectrum, which is almost absent in the spectrum of CAB. What is also possible to see, is that apart from the shift of the maxima in Amide I and II bands, a change in the band profile with a change in temperature occurs.

To further check which secondary structure is mainly responsible for the abovementioned trends, we performed deconvolution and fitting of the Amide I and Amide II region from 1707 to 1476 cm${}^{-1}$. The number of bands selected in the fitting procedure was done based on reported deconvolution of the Amide I band for carbonic anhydrase and the statistical result of the fitting [33]. For this purpose, GRAMS software was used. The complete analysis of the spectra of the samples at both temperature ends, i.e., 30 and 120 °C for CAB and CAB spectra obtained after subtraction of CAB@Ferria and Ferria spectra are given in the Supplementary Materials (Figures S1–S4). The maxima of the bands and their relative percentage of integrated band areas are given in Table 2. The samples selected were the ones at both temperature ends, i.e., native CAB recorded at 30 °C (30CAB) and 120 °C (120CAB), and CAB spectra obtained after subtraction of CAB@Ferria and Ferria spectra recorded at 30 °C (30C@F-F) and 120 °C (120C@F-F). The assignment was performed according to Ref. [33]. For all the fits, the R${}^{2}$ > 0.999.

The percentage of the band area was calculated by taking the ratio of a particular band area obtained from the fitting procedure and the sum of the integrated intensities of all the bands in question (Amide I and II regions).

From Table 2 it can be seen that the main percentage of the intensity is due to the β-structure band (≈1630 cm${}^{-1}$) in the Amide I wavenumber region. As it can be seen, there is a blue shift of this band for CAB and C@F-F spectra, in accordance with the previously stated trend. The red-shift of the Amide II band in raising the temperature follows the trend of the component band at ≈1535 cm${}^{-1}$, which carries the largest percentage of the intensity. From the data in Table 2, one can also see that the appearance of the previously mentioned shoulder in the spectrum of C@F-F spectrum at 120 °C is due to the lowest frequency component of the Amide II band, which doubles its intensity percentage, compared to the CAB spectrum at 120 °C. This can be also seen from the spectra in the Supplementary Materials (Figures S1–S4).

**Figure 5:**
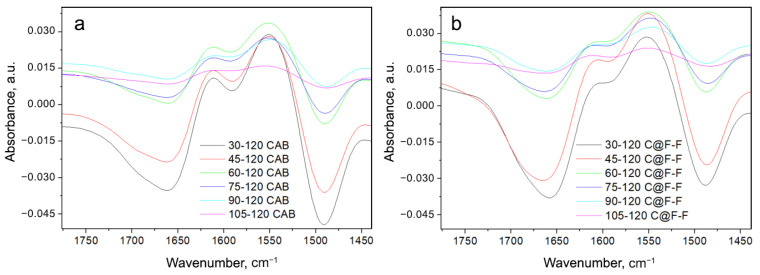
Difference spectra for CAB (**a**) and C@F-F (**b**), indicated through the label. Each spectrum is obtained as a difference between the spectrum obtained at a particular temperature and the one at 120 °C.

To see which of the bands that the Amide I and Amide II are composed of primarily change with the temperature change, we subtracted the spectra of CAB at any temperature with the spectrum recorded at 120 °C. This was also done for the C@F-F spectra, where each spectrum was subtracted from the one at 120 °C. The difference spectra are presented in Figure 5. Because the difference spectrum has a very low signal/noise ratio, we performed smoothing of the spectral lines.

From the change in the different intensities of C@F-F spectra (Figure 5a), it can be seen that the largest change occurs for the band minimum at 1657–1664 cm${}^{-1}$, is in connection to the melting of the α-helix structure [32]. Also, other structures, like bends and turns fall into this wavenumber region (Table 2). On the band maximum (or better, shoulder) at 1601–1614 cm${}^{-1}$ (band position at the lowest temperature and the highest temperature correspondingly), is less pronounced. This change is mainly due to β-structure but might also include a change in water and the side chain.

The changes in the difference spectra of CAB (Figure 5b) are more or less the same, except that changes of the 1604 cm${}^{-1}$ band are more pronounced and is visible in each difference spectrum. This is supported by the data presented in Table 3 for CAB spectra recorded at 30 and 120 °C, where one can see that the percentage of β-structure lowers with the increase of temperature, but this lowering is particularly pronounced for the helix structure of CAB. Another spectral characteristic in the CAB spectra is the appearance of another minimum (a shoulder) at 1700 cm${}^{-1}$ (Figure 5a).

**Figure 6:**
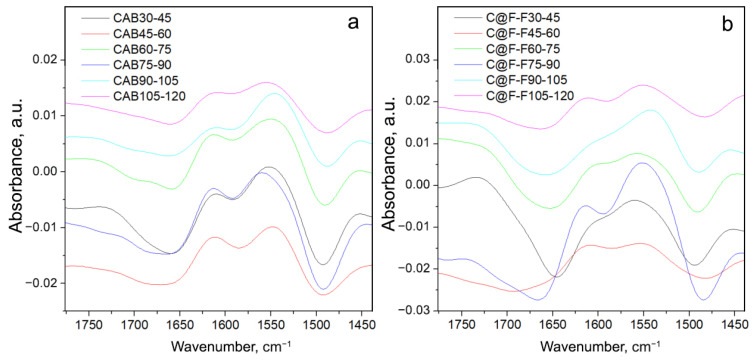
Difference spectra for CAB (**a**) and C@F-F (**b**). Each spectrum is obtained as the difference between the spectrum obtained at lower and the nearest higher temperature.

It is also possible to check the rate in the changes of the Amide I and II bands with the temperature if the difference between the spectra obtained at lower and the nearest higher temperature is calculated, as presented in Figure 6.

It can be seen from Figure 6 that the greatest change of the previously discussed band minima and maxima given in Figure 5 and Table 3, occurs for the spectrum C@F-F75-90. This is not a coincidence, as, at the temperature of 87 °C, the maximum in the cooperative melting of CAB was detected, for CAB in acidic D${}_{2}$O solution [32]. Thus, the changes in the α-helices and β-structures will be the greatest for spectra recorded at temperatures before and after the maximum structural change. Particularly visible is the change of the β-structure, through the appearance of the max. at 1616 cm${}^{-1}$, which is barely visible in the C@F-F30-45 and C@F-F45-60. On the other hand, there is a continuous change in the case of CAB. Thus, a similar effect may occur for CAB trapped in magnetite, i.e., a formation of a molten-globule state. This is further supported by the irregular changes in the C@F-F30-45 and C@F-F45-60, as in the temperature range 10–67 °C a non-cooperative melting of the secondary structure might have occurred as reported for the case of acidic CAB [32]. The data presented in Table 3 for the structural change of C@F-F also support these findings, where stark and unusual differences, for the integrated intensities of both α-helix and β-structure, are present between C@F-F samples for the spectra recorded at 30 and 120 °C. What is also interesting, inspecting the data in Table 2 is that relatively, β-structure presence in C@F-F is larger than in the native CAB, while oppositely is valid for the helix structure.

**Figure 7:**
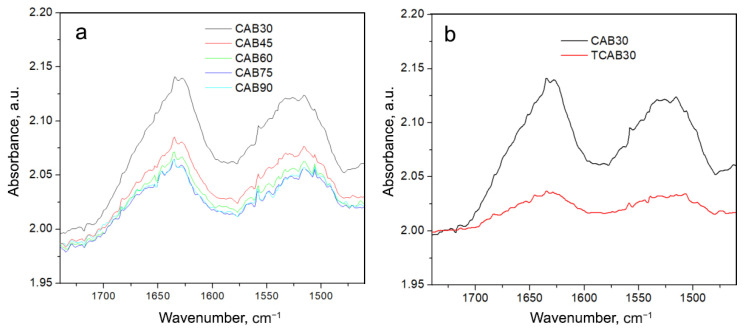
(**a**) Spectra of CAB recorded at different temperatures (from 30 to 90 °C with a step of 15 °C) in the region of Amide I and II vibrations. (**b**) Spectrum of CAB recorded at 30 °C (black curve); spectrum of CAB obtained at 30 °C after cooling from 90 °C (red curve).

**Figure 8:**
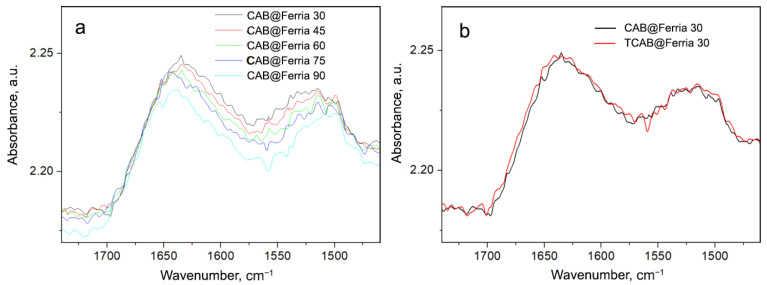
(**a**) Spectra of CAB@Ferria recorded at different temperatures (from 30 to 90 °C with a step of 15 °C) in the region of Amide I and II vibrations. (**b**) Spectrum of CAB@Ferria recorded at 30 °C (black curve); spectrum of CAB obtained at 30 °C after cooling from 90 °C (red curve).

To investigate the stability of CAB and CAB@Ferria samples, we increased the temperature to 90 °C (the temperature at which the activity may still be retained). The recorded spectra of these samples with the rise of the temperature are presented in Figure 7 and Figure 8.

From Figure 7 it is easily seen that there is a change in band intensities and their form with temperature, but more important is that after cooling down to 30 °C, there is a big difference between that spectrum and the originally recorded CAB spectrum at 30 °C (Figure 7b).

Again, the spectrum of CAB@Ferria changes with temperature as previously explained (Figure 8a). However, when comparing the spectra of CAB@Ferria originally recorded at 30 °C (Figure 8b, black curve) with the spectrum recorded at 30 °C after cooling from 90 °C, it is obvious that there is no difference between the bands either in intensity or in the form. This means that the structure of the CAB@Ferria changes with temperature, and so does its activity, but after cooling the CAB@Ferria returns to its original structure, i.e., the process is reversible, which couldn’t be said for CAB. This reversibility is characteristic of the formation of a molten-globule state [32].

## 3. Discussion

The problem of enzyme immobilization within sol-gel matrices is under intense investigation, and the list of the described systems is rising every day. The main attention is paid to silica-based biomaterials where new synthetic methodologies of entrapment are been developed, enzymatic activities are been analyzed and stability studies are been carried out [38]. The advantage of such materials was demonstrated in numerous studies, where silica sol-gel systems not only preserved the activity of the bioactive dopants but also provided exceptional chemical and thermal stability to a variety of proteins, enzymes, and even whole cells against various deterioration processes [39,40,41,42]. The attention towards silica materials is mainly dictated by their relative cheapness, convenience, and simple synthetic conditions, but alternative materials such as alumina, titania, or iron oxide-based systems can also be used for this purposes [43,44,45,46]. In our work, we used the original approach toward entrapment of biomolecules within sol-gel magnetite matrix at mild conditions based on readily available water-based magnetite hydrosol. The hydrosol was synthesized without any surfactants to make it bio-friendly by design to prevent premature denaturation of the added biomolecules. The absence of surfactants was also important for the possibility of sol-gel transition iiit the system, which is not possible in the case of modified surfaces.

The exceptional thermal stability of entrapped enzymes has been demonstrated by many research groups and has generally been associated with the intimate interaction of entrapped enzymes with the three-dimensional sol-gel matrix structure within a single pore and surface-bound water molecules retaining the secondary structure of the trapped enzymes [17,47,48]. It is widely accepted that maintaining good hydration of the sol-gel matrix by mild synthetic conditions and in some cases application of osmolytes play a key role in the stability of bio-doped ceramics even at high temperatures [49]. In the case of CAB@Ferria composite suitable hydration of the entrapped enzyme is maintained due to surface-bound water molecules whose IR bands can be observed even at 200 °C [50].

Since the structural integrity of biomolecules determines their bioactivity, this problem has been investigated in a variety of ways that describe the structural properties of biomolecules and their rearrangements in response to external stimuli [18,35,51,52]. Unfortunately, in the case of Ferria doped materials one of the most powerful techniques namely circular dichroism is unsuitable due to strong UV adsorption by magnetite nanoparticles, thus it was investigated via ATR-FTIR spectroscopy.

IR-ATR investigations of native CAB and CAB@Ferria showed differences in their structural behavior with the temperature change. In both cases, there is a shift of the Amide I to higher and Amide II to lower frequencies with an increase in the temperature. The reason for this behavior is probably due to the weakening or breaking of hydrogen bonds in the protein. The deconvolution and fitting of the Amide I and II bands also confirm that the main component of the Amide I band (β-structure band at 1630 cm${}^{-1}$), is also blue shifted. The red-shift of the Amide II band maximum is due to the red-shift of the 1535 cm${}^{-1}$ component. The difference between spectra recorded at lower temperatures and the one at 120 °C, reveals that the main change occurs for the helix structure, while this change of the β-structure is much lower for the C@F-F than for CAB, which might be in connection to the non-cooperative and cooperative melting of C@F-F and CAB, respectively. That a formation of a so-called molten-globule state for CAB caged in Ferria might be the case, is supported by the difference in the spectra recorded at neighboring temperatures. In the case of CAB, there is a regular change of the difference bands, while in the case of C@F-F there is an abrup tion from the regularity for the difference spectrum C@F-F75-90. The reason for that might be that this difference spectrum reflects the change from non-cooperative, to cooperative melting. That this molten-globule state of the CAB embedded in Ferria NP can be a possibility, is depicted through the measurement of the spectrum of CAB and C@F-F up to 90 °C, and then cooling back to 30 °C. The spectrum of CAB was completely changed, with diminished Amide I and II band intensities, while C@F-F reversed back to the spectrum characteristic for 30 °C.

## 4. Materials and Methods

*Chemicals:* Carbonic anhydrase from bovine erythrocytes (CAB, cat. # C-2624), Trisma hydrochloride, sodium hydroxide, iron (II) chloride tetrahydrate, iron (III) chloride hexahydrate, and ammonia were all obtained from Sigma-Aldrich.

*Ferria hydrosol:* Ferria hydrosol was prepared by the procedure described earlier [5,53]. Briefly, 2.5 g of iron (II) chloride tetrahydrate and 5 g of iron (III) chloride hexahydrate were dissolved in 100 mL of deionized water and 11 mL of ammonia was added rapidly under constant stirring. The magnetic phase was magnetically separated and washed with water until a neutral pH level. After washing magnetic nanoparticles were dispersed in 100 mL of water and ultrasonically threatened for two hours.

*Synthesis of CAB@Ferria:* The composite was produced by the procedure described in Ref. [23] Briefly, 200 μL of Ferria was mixed with 20 μL of CAB solution 10 g/L in 0.05 M Tris buffer at pH 7.4 and dried under vacuum at 20 °C. The resulting composite was washed with Tris buffer and dried.

*Enzymatic assay:* 1 mg of free CAB or equivalent mass of CAB@Ferria was dissolved in 2 mL of Tris buffer (pH 7.4) and incubated at the desired temperature for 10 min. After that, 125 μL of pNPA solution (0.8 M in acetone) was added, and enzymatic activity was spectrophotometrically measured at 405 nm in thermostatic conditions. As the control, the rate of pNPA self-hydrolysis alone and in the presence of an empty Ferria sol-gel matrix was measured and subtracted from the experimental results.

*IR-ATR spectroscopy:* The IR-ATR spectra were recorded on a Perkin-Elmer FT-IR System 2000 (USA) using Temperature controlled Golden Gate ATR accessory (product of Specac, UK), equipped with a diamond ATR crystal. The ZnSe lenses allowed ATR spectra to be recorded as low as 520 cm${}^{-1}$. The spectra of the samples (CAB, CAB@Ferria, and Ferria) and the background (air/nitrogen) were recorded using 64 scans and 4 cm${}^{-1}$ resolution. Because the index of refraction of a medium depends on the temperature, the single-beam spectra were recorded at temperatures of 30, 45, 60, 75, 90, 105, and 120${}^{\circ }$ in a row, for that background first, and then the sample spectra were recorded under the same conditions, without removing the sample. Around 10 min between the attained temperature and the corresponding measurement was allowed to pass, in order for the sample to obtain the correct temperature and changes in it due to the temperature occurred. The corresponding sample and background spectra (recorded at the same temperature) were afterward divided in order to obtain the ATR spectra of the samples. The sample compartment and detector were purged with nitrogen with 99.999% purity.

## 5. Conclusions

The two-component enzymatically active magnetic composite was synthesized via the sol-gel method using bio-friendly magnetite hydrosol under mild conditions by entrapment of CAB. The material showed exceptional thermal stability and was active at temperatures exceeding the denaturation temperature of the free enzyme for at least 20 °C. In this work, we made a first attempt to describe the observed stability of the entrapped enzyme by analyzing its secondary structure in response to thermal shock by using FTIR spectroscopy. The obtained results may be used as a basis for the development of bioactive magnetic ceramic materials with improved thermal stability.

## Figures and Tables

**Table 1:** pNPA conversion rate as a function of temperature for CAB and CAB@Ferria.

Temperature, °C	CAB, μM/min	CAB@Ferria, μM/min
20	3.30	0.27
30	4.18	0.56
40	4.80	0.87
50	4.66	1.30
60	2.80	1.82
65	0.78	1.79
70	0	1.62
80	0	1.30
90	0	0.79
95	0	0.45

**Table 2:** Wavenumber maxima in cm${}^{-1}$ and band areas in percentage from the deconvoluted bands of Amide I and Amide II.

Sample	Turns, Bends	β-Structure	Turns, Bends	Turns, Bends	α-Helix	β-Structure	H${}_{2}$O, (Side Groups)	Amide II		
	ν/cm${}^{-1}$									
30CAB	1695	1681	1671	1664	1656	1628	1593	1536	1511	1496
120CAB	1696	1682	1679	1666	1655	1632	1601	1533	1510	1495
30C@F-F		1685	1675	1666	1655	1631	1592	1539	1510	1495
120C@F-F	1707, 1695	1682		1666	1651	1632	1598	1537	1513	1496
	Band integrated area/%									
30CAB	0.93	3.15	3.08	1.18	10.28	26.88	19.15	22.91	10.85	1.59
120CAB	1.06	1.36	5.38	2.97	8.15	26.59	17.25	20.18	11.77	5.29
30C@F-F		3.03	2.71	3.48	3.63	33.48	17.71	20.84	12.10	3.01
120C@F-F	2.00	4.44		8.06	5.18	28.97	15.05	14.20	11.44	10.68

**Table 3:** Minima and maxima in the difference spectra of CAB and C@F-F. The difference spectrum is between the spectrum at a particular temperature and the one recorded at 120 °C.

Position	Min	Min	Max	Min	Max	Min
ν(CAB)/cm${}^{-1}$	1700	1661	1611	1591	1550	1490
ν(C@F-F)/cm${}^{-1}$		1663	1604	1594	1551	1487

## Data Availability

Not applicable.

## Supplementary Materials

The following supporting information can be downloaded at: https://www.mdpi.com/article/10.3390/ijms23115975/s1.

## Abbreviations

The following abbreviations are used in this manuscript:

CAB	Carbonic anhydrase from bovine erythrocytes
NPs	nanparticles
DCS	dynamic scanning calorimetry
FTIR	Fourier-transform infrared spectroscopy
ATR	Attenuated total reflection
pNPA	para-nitrophenylacetate
