# Supplementary material for: Structural Rearrangements of Carbonic Anhydrase Entrapped in Sol-Gel Magnetite Determined by ATR–FTIR Spectroscopy
Source: Int J Mol Sci. 2022 May 26;23(11):5975. doi: 10.3390/ijms23115975 (PMC9181146; doi:10.3390/ijms23115975)
Supplement: Supplementary file 1 [file ijms-23-05975-s001.zip › ijms-1717337-supplementary.pdf]

Supplementary Materials

Structural Rearrangements of Carbonic Anhydrase Entrapped in Sol-Gel Magnetite Determined by ATR–FTIR Spectroscopy

Vladimir Ivanovski [1,*], Olga Shapovalova [2] and Andrey S. Drozdov [3]

[1] Faculty of Natural Sciences and Mathematics, Institute of Chemistry, Ss. Cyril and Methodius University in Skopje, Arhimedova 5, 1000 Skopje, North Macedonia

[2] SCAMT Institute, ITMO University, Lomonosova St. 9, 191002 Saint Petersburg, Russia; shapovalova@scamt-itmo.ru

[3] Moscow Institute of Physics and Technology, Institutsky Ave. 9, 141701 Dolgoprudny, Moscow Region, Russia

* Correspondence: vladimir.ivanovski@yahoo.com (V.I.); drozdov.science@gmail.com (A.S.D.)

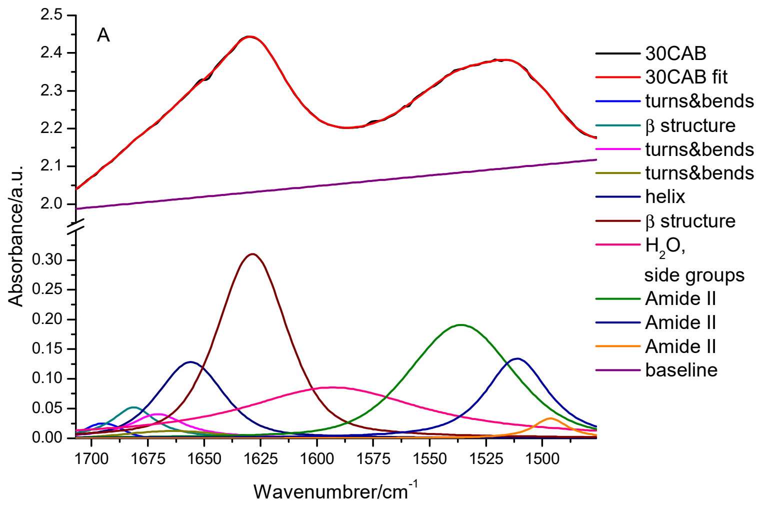

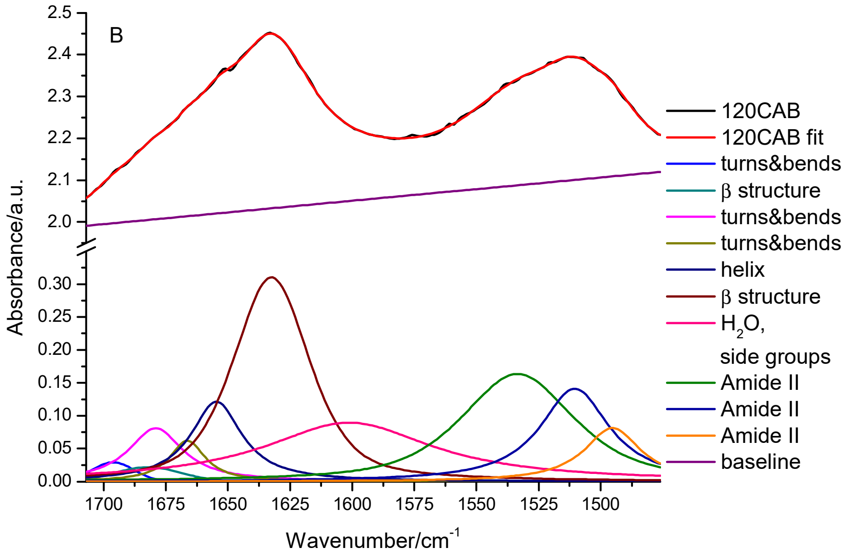

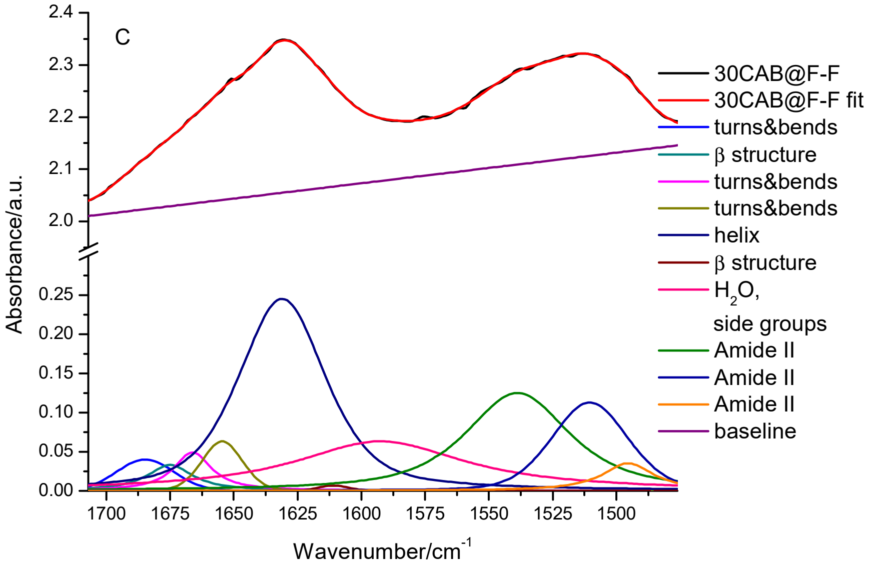

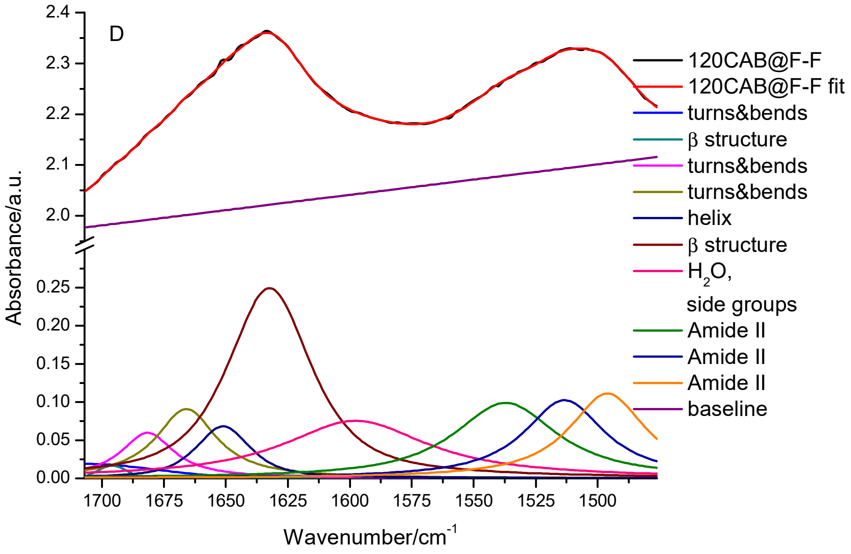

Figure S1. Deconvoluted and fitted spectrum of: native lyophilized CAB recorded at 30 °C (A), native lyophilized CAB recorded at 120 °C (B), CAB spectra obtained after subtraction of CAB@ferria and ferria spectra recorded at 30 °C (C), CAB spectra obtained after subtraction of CAB@ferria and ferria spectra recorded at 120 °C (D). The data on the integrated intensities is given in Table 2. .
